# Feasibility, Adherence, and Effectiveness of Blended Psychotherapy for Severe Mental Illnesses: Scoping Review

**DOI:** 10.2196/43882

**Published:** 2023-12-26

**Authors:** Yamina Ehrt-Schäfer, Milan Rusmir, Johannes Vetter, Erich Seifritz, Mario Müller, Birgit Kleim

**Affiliations:** 1 Department of Psychology University of Zurich Zurich Switzerland; 2 Department of Psychiatry, Psychotherapy and Psychosomatics, Psychiatric Hospital University of Zurich Zurich Switzerland

**Keywords:** blended psychotherapy, severe mental illnesses, digital health intervention, e-mental health, scoping review

## Abstract

**Background:**

Blended psychotherapy (bPT) combines face-to-face psychotherapy with digital interventions to enhance the effectiveness of mental health treatment. The feasibility and effectiveness of bPT have been demonstrated for various mental health issues, although primarily for patients with higher levels of functioning.

**Objective:**

This scoping review aims to investigate the feasibility, adherence, and effectiveness of bPT for the treatment of patients with severe mental illnesses (SMIs).

**Methods:**

Following the PRISMA-ScR (Preferred Reporting Items for Systematic Reviews and Meta-Analyses Extension for Scoping Reviews) guidelines, we conducted searches in PubMed, MEDLINE, Embase, PsycINFO, and PsycArticles for studies published until March 23, 2023.

**Results:**

Out of 587 screened papers, we incorporated 25 studies encompassing 23 bPT interventions, involving a total of 2554 patients with SMI. The intervention formats and research designs exhibited significant variation. Our findings offer preliminary evidence supporting the feasibility of bPT for SMI, although there is limited research on adherence. Nevertheless, the summarized studies indicated promising attrition rates, spanning from 0% to 37%, implying a potential beneficial impact of bPT on adherence to SMI treatment. The quantity of evidence on the effects of bPT for SMI was limited and challenging to generalize. Among the 15 controlled trials, 4 concluded that bPT interventions were effective compared with controls. However, it is noteworthy that 2 of these studies used the same study population, and the control groups exhibited significant variations.

**Conclusions:**

Overall, our review suggests that while bPT appears promising as a treatment method, further research is necessary to establish its effectiveness for SMI. We discuss considerations for clinical implementation, directions, and future research.

## Introduction

Mental illnesses are widespread globally, with estimated lifetime prevalence rates ranging from 12% to 47% [1,2]. There is a potential increase in these rates [3,4], placing a substantial burden on individuals [5,6], their families [7], and both economic and public health systems [8]. Psychotherapy stands as an evidence-based primary treatment for the majority of mental disorders [9], as advocated by both national and international guidelines, such as those from the American Psychological Association (APA) and the National Institute for Health and Care Excellence (NICE). Blended psychotherapy (bPT), alternatively termed “blended therapy” or “blended psychological treatment,” involves combining conventional face-to-face therapy with digital intervention components within a unified treatment protocol. This integration aims to amplify the efficacy and cost-effectiveness of mental health care [9-11]. In recent years, the literature has detailed various modes of bPT, encompassing integrated use (where face-to-face and digital components are concurrently administered) and sequential use (where the digital intervention takes place either before or after the face-to-face component) [11]. From a conceptual standpoint, Bielinski and colleagues [10] make a further distinction between transformational blends (using digital and face-to-face components in an interconnected manner) and additional blends (involving more independent combinations of digital and face-to-face therapy components).

Recent research offers evidence supporting the feasibility and effectiveness of bPT when compared with control conditions [11]. This holds true across a spectrum of mental health conditions, including depression, anxiety disorders, and substance abuse, and is applicable to both adult and adolescent patients [11-13]. bPT offers several advantages and potentials in comparison to conventional face-to-face psychotherapy. These include heightened exposure to treatment within and beyond the therapy session, fostering patient empowerment in self-management [14], improved consolidation of therapy content [15], enhanced transfer of in-session outcomes into everyday life [16], and the sustenance of therapeutic changes beyond the acute treatment phase. Moreover, bPT has the potential to contribute to the development and stabilization of the therapeutic relationship over time [11,17]. For therapists, the benefits of using bPT are potential time savings [11] and the capacity to customize treatments more flexibly according to patients’ needs. Furthermore, the incorporation of digital data into the bPT intervention, whether actively (eg, through direct symptom monitoring, ecological momentary assessment) or passively (eg, via wearable devices and device metrics), grants therapists access to detailed patient data. These data can be instrumental in enhancing treatment through feedback mechanisms and early detection of crises or emergency situations [18-20].

Numerous studies have concentrated on patients with relatively high levels of functioning [13,21]. Nevertheless, there is limited research on the feasibility and effects of bPT for patients with severe mental illnesses (SMIs) [12,13,22,23], even though there is mounting evidence regarding the effectiveness of psychotherapy for this population [24-28]. Individuals with SMI, marked by considerable limitations in intrapersonal and social functioning [24,29], frequently necessitate long-term, intensive, multidisciplinary treatment and care, which can be financially burdensome [30,31]. These patients, with an estimated annual prevalence rate of 2.33 per 1000 for all disorders [32], frequently make up the majority of patients in psychiatric hospitals and long-term psychiatric care. Consequently, our objective is to consolidate the current evidence on the feasibility and effectiveness of bPT for SMI. In particular, we aim to scrutinize the characteristics of bPT interventions implemented for SMI patient groups, examining aspects such as feasibility, adherence, and effectiveness.

## Methods

### Study Design

We performed a scoping review, following the framework stages proposed by Arksey and O’Malley [33] and further refined by Levac and colleagues [34,35]. The reporting adhered to the PRISMA-ScR (Preferred Reporting Items for Systematic Reviews and Meta-Analyses Extension for Scoping Reviews) guidelines [36] (Multimedia Appendix 1). This review approach is designed to address an exploratory research question with the aim of mapping the existing literature and key concepts in a defined field. It serves to identify gaps and types of evidence in research [35].

### Search Strategy

We performed a systematic search of the PubMed, MEDLINE, Embase (via Elsevier), PsycINFO, and PsycArticles databases. The search terms included the following keywords: (1) treatment ((blended) AND (therapy)); (2) broader digital health ((internet) OR (mobile) OR (online) OR (digital)); and (3) population (“severe mental illness” OR “serious mental illness” OR “schizophrenia” OR “psychosis” OR “psychotic” OR “psychotic disorder*” OR “schizoaffective” OR “schizo-affective” OR “bipolar disorder*” OR “mania” OR “manic” OR “bipolar” OR “depression” OR “major depressive disorder” OR “antipsychotic” OR “personality disorder” OR “inpatient”). The search strings were converted for each database. We also manually searched the bibliographies of relevant studies and reviews using the snowball principle to identify further eligible articles. We included publications from the date of inception up to March 23, 2023, in the search.

### Study Selection

#### Eligibility Criteria

Studies were included if they met the following criteria: (1) peer-reviewed articles written in English; (2) had a focus on blended psychotherapeutic study interventions (ie, a combination of face-to-face psychotherapy treatment with online, mobile, or other digital content [10]); (3) inclusion of a study population with severe psychiatric disorders. SMI could be defined by the study author; a psychiatric diagnosis reflecting common definitions of SMI [29,32], such as schizophrenia spectrum disorders or other psychotic disorders, bipolar disorders, severe depression, or severe personality disorders; severity of a diagnosis, indicated by psychometric measures (eg, Beck’s Depression Inventory Second Edition [BDI-II]≥30; 9-item Patient Health Questionnaire [PHQ-9]≥20; Generalized Anxiety Disorder [GAD]≥15); or inpatient or previous inpatient treatment setting (indicating greater functional impairment and severity). Articles reporting on (4) feasibility or adherence or efficacy or effectiveness of the respective bPT intervention were included.

Because of the novelty of the field, there was no restriction on the variety of study designs. However, we excluded articles on digital stand-alone interventions, as well as those on digital therapist-assisted (guided) interventions that lacked face-to-face therapist-patient contact. Additionally, e-mental health applications solely focused on symptom monitoring (ambulatory assessment) without a psychological intervention component were excluded.

#### Study Selection Process

The relevant research papers that were identified underwent screening based on information available from the study title and abstract. One reviewer (MR) piloted a study screening manual, which was subsequently discussed and further refined by the study team through group consensus. Abstracts and full-text articles were independently reviewed by 3 reviewers (MR, YE-S, and Lilian Fraefel). Any disagreements were resolved through discussions with the senior researcher (BK), who made the final decision regarding inclusion in cases of uncertainty.

### Quality Assessment

In accordance with the PRISMA-ScR guidelines for conducting scoping reviews [36,37], formal quality appraisal was not incorporated into the study selection process. Nevertheless, we evaluated the study quality of the selected articles using a quality assessment checklist developed by Ellis and colleagues [38]. This was done to ensure that the included studies adhered to standard research norms and to provide insights for future research. The scores of the studies ranged from 9 to 11 (out of a maximum of 12), signifying a high level of quality for the selected articles. Further details can be found in Multimedia Appendix 2 [39-61].

### Data Charting

Data charting was undertaken as a collaborative and iterative process. A customized data extraction worksheet was created and consistently adjusted to capture all data that could be pertinent to addressing the outlined research questions. The ultimate data chart underwent scrutiny for validity and data accuracy by 6 members of the research team. The comprehensive data extraction form is available in this publication (refer to Multimedia Appendix 3 [30,39-62]).

## Results

### Study Selection

**Figure 1:**
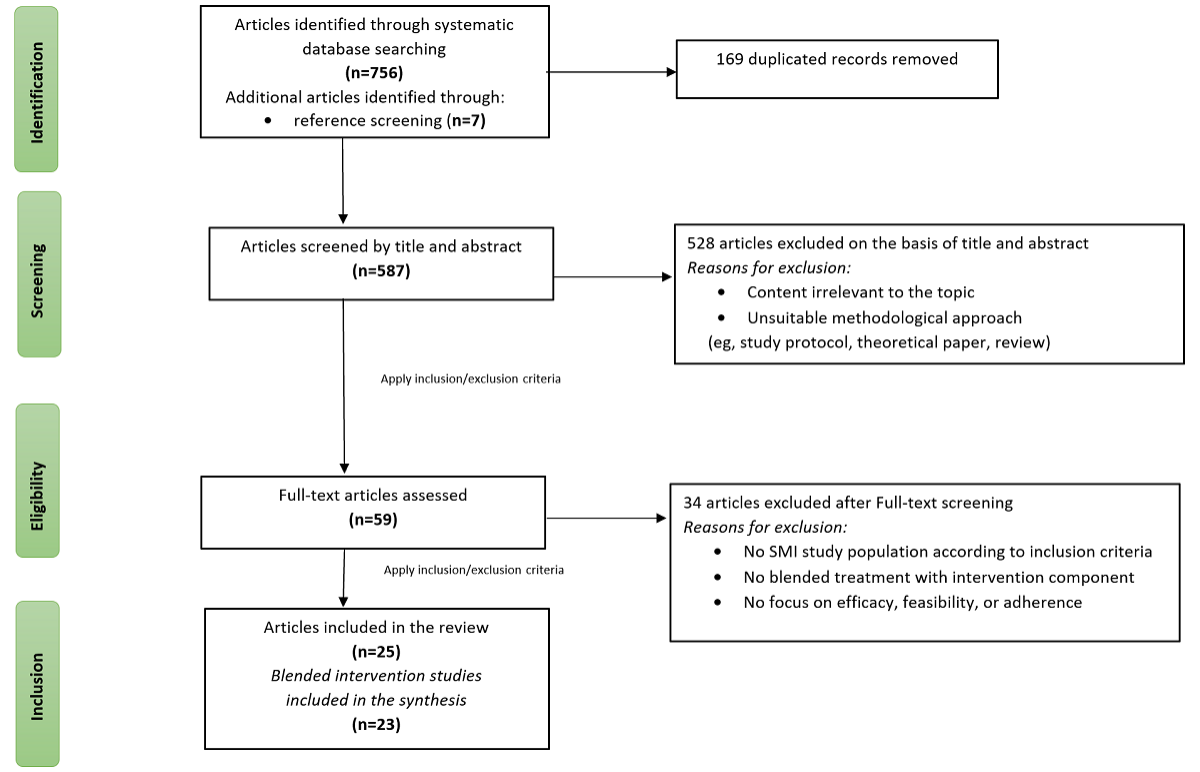
PRISMA flow diagram. The overall process of article selection following PRISMA-ScR guidelines. PRISMA: Preferred Reporting Items for Systematic Reviews and Meta-Analyses; PRISMA-ScR: PRISMA Extension for Scoping Reviews; SMI: severe mental illness.

Following the removal of duplicates, a total of 587 articles underwent screening, and 59 papers were assessed in full for eligibility. We identified 25 articles that were relevant to the scope of the review. Among these, 23 papers [39-61] reported as main studies on aspects of feasibility, adherence, and effectiveness of bPT interventions, while 2 [30,62] were substudies analyzing aspects of adherence only. For the quantitative analysis of the review, these 2 substudies will not be included. We specifically utilized the information for the analysis of adherence. The outcomes of the article selection process are illustrated in Figure 1.

### Study Characteristics

Tables 1 and 2 outline the characteristics of the included studies (n=23) and patients (n=2554). The papers incorporated into the review were predominantly from research groups in Germany (8/23, 35%) [43,44,46-48,53,57,58], followed by the United Kingdom (5/23, 22%) [39,40,51,54,59], the Netherlands (4/23, 17%) [49,50,55,61], and Australia (3/23, 13%) [42,45,56]; 1 study was conducted in the United States [60], and 2 were structured as transnational collaborative research [41,52]. We identified articles published between 2012 and 2021.

Among the bPT studies, the majority (12/23, 52%) utilized randomized controlled trial designs [43-49,51,52,57,59,61]; 2/23 studies (9%) were nonrandomized controlled trials [42,55], and 1 used a quasi-experimental 4-group design [39]. The remaining studies comprised 6/23 (26%) observational pilots [50,53,54,56,58,60], 1 observational study [40], and 1 qualitative evaluation [41].

Among the bPT trials with control groups (15/23; 65%) [39,40,42-49,51,52,57,59,61], 11 utilized treatment as usual (TAU) groups [40,45-49,51,52,57,59,61], 3 used an active control group [39,43,44], and 1 study had a waiting list control group [42]. The bPT interventions covered a range of SMI study populations, involving a total of 2554 patients with SMI. Notably, for the trials conducted by Zwerenz and colleagues [43,44], the same study population was utilized to generate different data sets—1 on inpatient treatment and another for follow-up on the same population after inpatient treatment.

**Table 1:** The main features of the blended psychotherapy studies included (n=23).

Article characteristics		Value, n
**Country of origin (n=23)**		
	Australia/United Kingdom	1
	Germany/Denmark	1
	United States	1
	Australia	3
	The Netherlands	4
	United Kingdom	5
	Germany	8
**Publication year (n=23)**		
	2012	1
	2013	2
	2016	3
	2017	2
	2018	4
	2019	2
	2020	3
	2021	6
**Research design (n=23)**		
	Randomized controlled trial	12
	Nonrandomized controlled studies	2
	Quasi-experimental	1
	Observational pilot	6
	Observational	1
	Qualitative evaluation	1
**Control group designs (n=15)**		
	Treatment as usual	11
	Active control group	3
	Waiting list	1

**Table 2:** Study populations.^a^

Patient demographics		Value (N=2554), n
**Disorder specific (n=14)**		
	Hoarding disorder	1
	Bulimia nervosa	1
	Anorexia nervosa	2
	Borderline personality disorder	2
	Major depression^a^	3
	Psychotic disorder	5
**Transdiagnostic/symptom oriented (n=8)**		
	Affective symptoms	3
	Psychotic symptoms	2
	Severe mental illness, various disorders^b^	3

^a^The study population of Zwerenz et al [43,44] counts as 1 population. The total is accordingly adjusted.

^b^Patients fulfilling SMI criteria according to [29].

We identified 14/22 (64%) disorder-specific study populations, comprising patients with psychotic disorders (n=5), severe major depression (n=3), borderline personality disorder (n=2), anorexia nervosa (n=2), bulimia nervosa (n=1), and hoarding disorder (n=1). Additionally, we identified 8/23 (35%) transdiagnostic or symptom-oriented bPT interventions: 3 targeting affective symptoms, 2 addressing psychotic symptoms, and 2 designed for patients meeting the criteria of SMI as defined by the Dutch consensus group on SMI [29].

### Characteristics of bPT Interventions

We identified a variety of bPT intervention formats (Tables 3-6), primarily focusing on outpatient treatment (21/23, 91%). The duration of the analyzed interventions ranged from 2 sessions to 18 months, varying based on the specific objectives of each intervention. Among the 23 interventions, 17 (74%) used an integrated blending approach [39,43-45,49-61], with 12 of them adopting a concept of transformational blending, and 5 [42,45-48] utilizing an additional blend. Additionally, 6/23 (26%) studies designed the bPT interventions in a sequential manner [40,42,45-48]. One study incorporated the digital component before intensive face-to-face psychotherapy [40], while 5 studies [42,45-48] included the digital component as part of an after-care concept following intensive face-to-face treatment. Among the sequentially designed interventions, 2 used a transformational blend [40,42], and 4 utilized an additional blend [45-48].

**Table 3:** Characteristics of bPT^a^ study interventions: Integrated use of digital and face-to-face components with complementary contents of both components (transformational blend).

Study	Project/digital component duration	Study population	Treatment setting	Psychotherapy treatment	Digital component and face-to-face component	Peer group
Beentjes et al [61]	e-IMR/12 months	SMI^b^; various disorders	OP^c^	IMR^d^/recovery	Digital: An online platform providing IMR content with added illustrative videos showing peer testimonials to encourage participants to talk more freely about themselves and to take steps in their recovery process; additionally, problem-solving forms at the end of each module, registration of successful coping strategies, and a symptom-monitoring page. Face-to-face: IMR-based care with standardized, curriculum-based group therapy providing psychoeducation on skills for illness management and recovery; additionally, extensive inpatient or outpatient psychiatric treatment including case management.	Yes
Bell et al [52]	SAVVy/3 weeks	Psychotic disorder	OP	CBT^e^/CSE^f^	Digital: usage of an existing smartphone app with EMA^g^ and EMI^h^; EMA included 10 surveys per day for 6 days; EMI included 5 coping reminders per day and evening survey with feedback for every day; EMI use was divided into 2 periods of 10 days each. Face-to-face: 4 sessions between EMA and EMI usage, discussion on EMA/EMI usage, app training and app coding, analyzing and further developing of coping strategies, and providing positive reinforcement.	No
Bendig et al [53]	Blended SST/4 weeks	SMI; various disorders	IP^i^	CBT/social skills	Digital: an online program with modules on social skills training, including psychoeducation and exercises on social competence/social skills. Face-to-face: group training on social competence/social skills.	No
Blankers et al [55]	Blended FACT^j^/Arkin mental health care/3 months	SMI; various disorders	OP	FACT/recovery	Digital: an online platform for i-FACT (flexible assertive community treatment), using the internet portal “myMentrum”; psychoeducation videos, leisure activity bulletin board, agenda for scheduling appointments with the psychiatric nurse; a web forum to establish contact with other patients; additional use of Skype for contact with psychiatric nurses (either scheduled or during office hours). Face-to-face: FACT.	Yes
Craig et al [59]	AVATAR/12 weeks	Psychotic disorder; auditory verbal; symptoms	OP	RA^k^/IPT^l^	Digital: computer-based AVATAR program with therapist facilitation of discussion between patient and avatar. Face-to-face: standard psychiatric care, including progress discussion after each session.	No
Garety et al [51]	SlowMo/12 weeks	Psychotic disorder; paranoia symptoms	OP	CBTp^m^	Digital: a web-based and mobile app with i-CBTp^n^; interactive features including psychoeducation, animated vignettes, games, and personalized content on coping with distressing paranoia symptoms. Face-to-face: digitally supported CBTp consisting of 8 individual, face-to-face sessions (60-90 minutes) in accordance with a clinical manual.	No
Granholm et al [60]	CBT2go/24 weeks	Psychotic disorder	OP	CBT/cognitive skills training	Digital: smartphone mobile app with cognitive skills content, personalized activity prompts, pleasure-savoring prompts (photos and journal), and reminder alerts for activities. Face-to-face: modified 12-session version of the Cognitive Skills Module of CBSST^o^, weekly group therapy sessions.	Yes
Jacob et al [58]	priovi/12 months	BPD^p^	OP	ST^q^	Digital: an online program with 8 ST-based modules; simulated dialogs via chatbot, daily SMS text messages, exercises; and a hotline for technical support. Face-to-face: weekly PT^r^ sessions.	No
Kooistra et al [49]	dCBT^s^ program/9 months	Severe depression	OP	CBT	Digital: an online program with modular CBT-based content, including psychoeducation, homework tasks/exercises, and an open-ended session evaluation for feedback on sessions and reflective thinking; and an additional email reminder to encourage patients to access the online platform. Face-to-face: weekly semistructured CBT-PT session.	No
Kooistra et al [50]	dCBT program/10 weeks	Severe depression	OP	CBT	Digital: an online program with modular CBT-based content, including psychoeducation, homework tasks/exercises, and an open-ended session evaluation for feedback on sessions and reflective thinking; and an additional email reminder to encourage patients to access online platform. Face-to-face: weekly semistructured CBT-PT sessions.	No
Sedgwick et al [54]	GRASP/4 weeks	Psychotic disorder	OP	CBT/social cognition training	Digital: a smartphone app with social cognition training content, including homework modules covering different topics and interactive features such as videos, games, and tasks. Face-to-face: social cognition training group therapy with an introduction to the homework app.	No
Thomas et al [56]	SMART/3 months	Psychotic symptoms	OP	Recovery	Digital: a website with a series of peer video interviews of people with lived experience of psychosis discussing how they had navigated issues within their own recovery and recovery modules including psychoeducation, additional reflective exercises, and self-monitoring. Face-to-face: 8 sessions with a mental health care worker, discussing contents of the website material.	No

^a^bPT: blended psychotherapy.

^b^SMI: severe mental illness.

^c^OP: outpatient.

^d^IMR: Illness Management and Recovery Program.

^e^CBT: cognitive behavioral therapy.

^f^CSE: coping strategy enhancement.

^g^EMA: ecological momentary assessment.

^h^EMI: ecological momentary intervention.

^i^IP: inpatient.

^j^FACT: flexible assertive community treatment.

^k^RA: relational approach.

^l^IPT: interpersonal therapy.

^m^CBTp: cognitive behavioral therapy for psychosis.

^n^i-CBTp: internet-based cognitive behavioral therapy for psychosis.

^o^CBSST: cognitive-behavioral social skills training.

^p^BPD: borderline personality disorder.

^q^ST: schema therapy.

^r^PT: psychotherapy.

^s^dCBT: digital cognitive behavioral therapy.

**Table 4:** Characteristics of bPT^a^ study interventions: integrated use of digital and face-to-face components with independent contents of both components (additional blend).

Study	Project/digital component duration	Study population	Treatment setting	Psychotherapy treatment	Digital component and face-to-face component	Peer group
Austin et al [41]	IMPACHS (Monsenso and Time 4U)/6 months	Psychotic disorder	OP^b^	CBTp^c^	Digital: 2 integrated mHealth components: (1) the Monsenso smartphone app for self-monitoring of mood and psychotic symptoms including a tool for self-reflection, a library function for psychoeducation, an action-plan function for customizable plans on coping strategies, tools to identify and describe factors that lead to symptom exacerbation; (2) Time 4U, an internet-based training and learning management system, comprising 8 interactive CBTp modules (texts, videos, tasks, and follow-up questions); additionally, an interface that enables clinicians to monitor and support both components. Face-to-face: CBTp-based assertive outreach; psychoeducation with a focus on symptom coping, recovery, and functioning facilitation.	No
Cardi et al [39]	VodCast/2 sessions	Anorexia nervosa	IP^d^/OP	CBT	Digital: vodcasts (a short video clip to encourage reappraisal and acceptance of food), auditory and visual imagery with background relaxation and soothing music, psychoeducational content, and motivational interviewing. Face-to-face: standard inpatient treatment (CBT)/standard outpatient treatment (unspecified).	No
Klein et al [57]	REVISIT-BPD/12 months	BPD^e^	OP	ST^f^	Digital: an online program comprising 8 modules with contents of scheme therapy, simulated dialogs via chatbot, daily SMS text messages, and exercises; and a hotline for technical support. Face-to-face: standard outpatient treatment (unspecified).	No
Zwerenz et al [44]	Deprexis/12 weeks	Severe depression	IP	CBT/psychodynamic psychotherapy	Digital: an online program comprising a web-based self-help program (CBT-based modules), conducted 2 h per week (scheduled). Face-to-face: standard inpatient treatment (psychodynamic).	No
Zwerenz et al [43]	Deprexis/6 months	Severe depression	OP	CBT	Digital: an online program comprising a web-based self-help program (CBT-based modules). Face-to-face: standard outpatient treatment (unspecified).	No

^a^bPT: blended psychotherapy.

^b^OP: outpatient.

^c^CBTp: cognitive behavioral therapy for psychosis.

^d^IP: inpatient.

^e^BPD: borderline personality disorder.

^f^ST: schema therapy.

**Table 5:** Characteristics of bPT^a^ study interventions: sequential use of digital and face-to-face components with complementary contents of both components (transformational blend).

Study	Project/digital component duration	Study population	Treatment setting	Psychotherapy treatment	Digital component and face-to-face component	Peer group
Duffy et al [40]	Silver Cloud iCBT^b^/undefined, depending on the waiting list	Severe affective symptoms	OP^c^/before face-to-face PT^d^	CBT/pretherapy support	Digital: an online platform with CBT-based modules, including tools for self-monitoring, behavioral activation, cognitive restructuring, and challenging core beliefs. Face-to-face: group therapy, face-to-face counseling, face-to-face CBT, or CBT delivered by a clinician via the internet after the online intervention.	No
Fitzpatrick et al [42]	HoPE/12 weeks of group therapy and an 8-week online program	Hoarding disorder	OP/after face-to-face PT	CBT/MT^e^/RP^f^	Digital: an online platform with 8 CBT-based modules, including worksheets and interactive content, homework for each week, and optional therapist–assisted email support (a maximum of 2 emails per week). Face-to-face: group therapy before the digital intervention.	No

^a^bPT: blended psychotherapy.

^b^iCBT: internet-based cognitive behavioral therapy.

^c^OP: outpatient.

^d^PT: psychotherapy.

^e^MT: maintenance therapy.

^f^RP: relapse prevention.

Concerning psychotherapeutic approaches, the majority of bPT interventions (16/23, 70%) used principles and methods derived from cognitive behavioral therapy (CBT) with various focuses. These ranged from disorder-specific CBT for depression [43,44,49,50], eating disorders [47,48,51], or psychosis [41,51,52] to competence training for social skills [53], social cognition skills [54], or voice coping [52]. Some interventions (4/23, 17%) centered on recovery [30,45,55,56] or maintenance therapy and relapse prevention [42,46-48]. Klein et al [57] and Jacob and colleagues [58] based their bPT interventions on the principles of schema therapy, and Craig and colleagues [59] used an interpersonal approach as the foundation of an AVATAR-based bPT program for the treatment of patients with SMI with psychotic symptoms [59]. Zwerenz and colleagues [43] integrated face-to-face psychodynamic inpatient treatment with a CBT-based online application (deprexis).

Among the 23 included bPT studies, a total of 20 different digital components were used. Some of the trials used the same digital component for different studies. Specifically, Jacob and colleagues [58] and Klein et al [57] used the priovi digital component, Kooistra and colleagues [49,50] used a self-developed i-CBT program, and Zwerenz et al [43,44] used the deprexis digital component.

The majority of the digital components (13/20, 65%) were based on online programs, while 5 (25%) used smartphone-based apps. One intervention incorporated an app that was compatible for both online and mobile use [51]. Two (10%) non-online–based digital components were included in the review. One study used vodcasts, which are short video clips aimed at encouraging reappraisal and acceptance of food in the blended treatment of patients with anorexia nervosa [39]. Another study combined an AVATAR program with face-to-face psychotherapy and psychosocial care to improve coping with distressing auditory verbal symptoms in psychosis [59].

A total of 7/35 (35%) digital applications provided opportunities for peer contact, either with known peers from the same treatment group [47,48,55,60] or with a broader peer community for mutual support and inspiration [30,45,46]. Therapist assistance for the digital component was implemented in 11/20 (55%) studies [41,42,45-50,55,58,61], either through individual feedback on patients’ completed assignments or by providing the option for patients to contact therapists via email or chat. Six applications offered electronic platforms or message boards for contacting and communicating with program peers [45-48,55,60], sometimes facilitated by a therapist (2/20, 10%) [47,48]. Tables 3-6 present a detailed account of the bPT intervention details.

**Table 6:** Characteristics of bPT^a^ study interventions: sequential use of digital and face-to-face components with independent contents of both components (additional blend).

Study	Project/digital component duration	Study population	Treatment setting	Psychotherapy treatment	Digital component and face-to-face component		Peer group
Alvarez-Jimenez et al [45]	Horyzons/18 months	Psychotic symptoms	OP^b^	Recovery/social functioning training	Digital: online platform based on the Moderated Online Social Therapy Model, interactive online therapy (“pathways” and “steps”), peer-to-peer online social networking, peer moderation, and expert support. Face-to-face: specialized care for early psychosis before the intervention and generic medical and mental health care according to individual needs.	Yes	
Ebert et al [46]	TIMT/12 weeks	Affective symptoms	OP/after IP^c^	CBT^d^/MT^e^	Digital: an online platform for peer support on the individual development plan, publishing weekly diary according to the individual development plan, and online peer support group. Face-to-face: standard inpatient treatment during hospital stay; independent treatment after discharge (unspecified).	Yes	
Fichter et al [47]	VIA/9 months	Anorexia nervosa	OP/after IP	CBT/MT/RP^f^	Digital: an internet platform with (1) modular psychoeducational content on anorexia nervosa, (2) a diary for self-monitoring, (3) a tool for structured functional behavioral analysis (SORC scheme), (4) an electronic message board for therapist-moderated peer support, and (5) regular email contact with the therapist. Patients’ platform usage is tracked to inform therapists. If the program is not used, then the therapist contacts the patient via phone or informs emergency contacts. Face-to-face: inpatient treatment during hospital stay and independent therapy after discharge (unspecified).	Yes	
Jacobi et al [48]	In-at-program/9 months	Bulimia nervosa	OP/after IP	CBT/MT/RP	Digital: an online platform including CBT-based content on bulimia nervosa with a moderated asynchronous online patient discussion group; personal mailbox; the opportunity to write down thoughts, to protocol own behavior including potential relapse trigger; CBT-based knowledge provision on prevention and self-management; 11 sessions; first 2 months a new session forth weekly, then 1 session per week on self-monitoring, psychoeducation, and peer-peer + patient-therapist communication. Face-to-face: standard inpatient treatment during hospital stay and independent treatment after discharge (unspecified).	Yes	

^a^bPT: blended psychotherapy.

^b^OP: outpatient.

^c^IP: inpatient.

^d^CBT: cognitive behavioral therapy

^e^MT: maintenance therapy

^f^RP: relapse prevention.

### Feasibility and Adherence

We defined feasibility as whether the bPT study intervention was suitable for the given setting and the respective study population. Adherence addresses participants’ engagement and compliance with the bPT treatment [62,63]. We found that 22/23 (96%) studies concluded that their bPT interventions were feasible. One study reported nonfeasibility due to participants’ lack of computer skills, trainers’ hesitation toward the program, and the program’s inflexibility [30]. Feasibility aspects focused on acceptability, patient and therapist satisfaction, and overall ratings, which were reported to be good to excellent (see Multimedia Appendix 3). Safety considerations were addressed in 7 studies [41,45,48,54,56,57,60], with 1 study [58] reporting adverse emotions triggered by self-use of the digital application.

Regarding treatment adherence, all but 1 study (22/23, 96%) reported on it, mainly focusing on patients’ adherence to the digital component. Various indicators were used, such as completed tasks, frequency and duration of usage, log-ins, self-reported usage, and participation in live chats. Four studies analyzed the impact of treatment adherence on intervention effects [47,48,58,60]. Two studies found no correlation [48,58], while 1 reported a positive association between app engagement and symptom reduction [60], and another identified a significant effect of treatment adherence on participants’ body weight [47].

Adherence to the face-to-face component was examined in 1 study [48], which found that patients without regular face-to-face treatment were more likely to not complete the online program. The overall treatment adherence rates varied between 0% and 37%, and in controlled trials, lower dropout rates were generally observed in the intervention treatment compared with control groups. In controlled trials, 12/15 (80%) reported lower dropout rates in the intervention treatment compared with control groups, although differences overall appeared small and heterogeneous, ranging between 0.5% [46] and 15% [55].

### Efficacy and Effectiveness of bPT for SMI

To provide a concise overview of the treatment effects of bPT interventions for SMI, the main outcomes of the controlled studies are summarized in Table 7.

Among the 15/23 (65%) studies that used control group designs to examine the efficacy or effectiveness of bPT interventions, 4 (17%) reported significant effects in favor of the intervention [43,44,46,59], with 2 of them using the same study population [43,44]. Three of these studies focused on bPT effects in patients with affective disorders [43,44,46], while Craig et al [59] investigated the treatment of distressing auditory verbal hallucinations as part of psychosis. Two trials compared bPT with TAU groups without a digital component [46,59], and Zwerenz et al [43,44] compared the intervention with an active control group with face-to-face treatment and different digital interventions.

Among the trials without significant main intervention effects, 5 reported significant effects on secondary outcomes [45,47,48,51,52], indicating potential efficacy. Klein et al [57], in a study involving patients with borderline personality disorder, found a significant intervention effect in a per-protocol analysis, specifically for participants from the intervention group who regularly used the digital component (priovi). Additionally, Cardi et al [39], in their quasi-experimental bPT study on the positive impacts of listening to vodcasts on food appraisal in patients with anorexia nervosa, found an interaction effect with the context: outpatients benefited more from the vodcast intervention, while inpatients reported more benefits from the control condition, which involved listening to classical music.

As many as 3/15 (20%) of the controlled studies [42,49,55] concluded that there were no group differences between intervention and control groups. Kooistra et al [49] in their investigation of the efficacy and cost-effectiveness of a manualized bPT intervention for patients with severe depression found no cost-effectiveness from a societal perspective but an acceptable probability of being cost-effective from the health care provider perspective.

Beentjes et al [61] reported significant effects in favor of the intervention for self-management, recovery, and general health perception. However, due to low treatment and study adherence, the authors deemed the results inconclusive.

**Table 7:** Main outcomes of controlled studies in the sample.

Study	Study design/mental health symptoms	Sample size, n	Control group	Efficacy/effectiveness
Ebert et al [46]	RCT^a^/affective symptoms	400	TAU^b^ included standard outpatient treatment without any digital component	The IG^c^ group was superior to the CG^d^; lower differences were observed in the change of general psychopathological symptom severity from discharge to 3- and 12-month follow-up in the IG. The IG exhibited less frequent symptom deteriorations and a higher frequency of remission at the follow-up assessments.
Zwerenz et al^e^ [44]	RCT/depression	229	The active CG received inpatient PT^f^ and psychoeducational information	The IG was superior to the CG; the IG demonstrated lower levels of depression and anxiety, along with higher quality of life and self-esteem, when compared with the CG.
Zwerenz et al^e^ [43]	RCT (follow-up)/depression	215	The active CG received outpatient PT and psychoeducational information	The IG was superior to the CG; the IG demonstrated lower levels of depression and anxiety, along with higher quality of life and self-esteem, when compared with the CG.
Craig et al [59]	RCT/psychotic disorder	150	TAU included supportive counseling without any digital component	The IG was superior to the CG; there was a reduction in the severity of auditory verbal hallucinations, but no effect on the malevolence of voices. At the 24-week follow-up, no significant differences were observed between the IG and the CG.
Alvarez-Jimenez et al [45]	RCT/psychotic symptoms	170	TAU included standard care and community mental health services	There were no group differences observed in the primary outcome of social functioning. However, in the IG, there were 5.5 times greater odds of finding employment or participating in an education program. By contrast, in the CG, there was a 2-fold increase in hospital admissions due to psychosis.
Bell et al [52]	RCT (pilot)/psychotic disorder	34	TAU included standard psychiatric care	There were no significant effects observed on the primary outcome of psychotic symptoms measured by PSYRATS^g^. However, the IG did show a significant effect, particularly in the Visual Analog Scale items related to coping with voices and awareness of patterns in voices.
Garety et al [51]	RCT/psychotic disorder	361	TAU included standard outpatient psychiatric care	There was no significant effect on the primary outcome of self-reported paranoia at 24 weeks compared with TAU. However, secondary beneficial effects on this measure were observed at 12 weeks. Both self-reported persecution and observer-rated paranoia showed improvement at both assessment points.
Fichter et al [47]	RCT/anorexia nervosa	258	TAU included standard outpatient treatment without any digital component	No group difference for the primary outcome general psychopathology; patients in the IG reported more weight gain than those in the CG.
Jacobi et al [48]	RCT/bulimia nervosa	253	TAU included standard outpatient treatment without any digital component	No group difference in abstinence rates; patients in the IG reported a lower frequency of vomiting than those in the CG.
Klein et al [57]	RCT/BPD^h^	204	TAU included standard outpatient treatment without any digital component	There was no significant effect in favor of the intervention in the overall analysis. The primary outcome, which measured the change in BPD symptoms (BPDSI^i^), decreased similarly in both groups. However, in the prespecified per-protocol analysis, which included only participants from the intervention group who used the intervention for at least 3 hours, a statistically significant intervention effect was found.
Cardi et al [39]	Quasi-experimental design/anorexia nervosa	38	The active CG received 4 pieces of classical music (20 minutes)	There was an interaction effect with the context. Specifically, outpatients derived more benefits from the vodcast, whereas inpatients experienced greater benefits from music. Interestingly, the vodcast did not lead to a reduction in distress and vigilance to food in the inpatient setting. Overall, there were no significant group differences between vodcast and music in the primary outcomes.
Fitzpatrick et al [42]	Pilot study; 2-group design/hoarding disorder	16	Waiting list	There were no group differences in the primary outcomes related to psychopathology. However, in the IG, there was a trend toward continued improvement in overall hoarding scores.
Blankers et al [55]	Pilot study; 2-group design/various disorders and SMI^j^	47	TAU included FACT^k^ without any digital component	There were no group differences observed, with both the IG and the care-as-usual group showing comparable improvements in quality of life and self-efficacy beliefs regarding their mental health problems. Additionally, HONOS^l^ scores did not change over time.
Kooistra et al [49]	RCT/depression	102	TAU included standard CBT^m^ without any digital component	There were no group differences observed for depressive episodes, response to treatment, and quality-adjusted life years. The intervention did not demonstrate cost-effectiveness from a societal perspective. However, there was an acceptable probability of being cost-effective from the health care provider’s perspective.
Beentjes et al [61]	RCT/SMI and various disorders	60	TAU included IMR^n^ without any digital component	There were significant effects in favor of the intervention for self-management, recovery, and general health perception. However, the results on effectiveness are inconclusive due to confounding factors and interaction modifications attributed to low study adherence.

^a^RCT: randomized controlled trial.

^b^TAU: treatment as usual

^c^IG: intervention group.

^d^CG: control group.

^e^bPT interventions with the same study population but different data.

^f^PT: psychotherapy.

^g^PSYRATS: Psychotic Symptom Rating Scales.

^h^BPD: borderline personality disorder.

^i^BPDSI: Borderline Personality Disorder Severity Index.

^j^SMI: severe mental illness.

^k^FACT: flexible assertive community treatment.

^l^HONOS: Health of the Nation Outcome Scale.

^m^CBT: cognitive behavioral therapy.

^n^IMR: Illness Management Recovery Program.

## Discussion

### Principal Findings

In this scoping review, we provided a comprehensive summary of the current evidence on bPT for SMI, with a specific emphasis on feasibility, adherence, and effectiveness. Our review encompassed 25 research papers, consolidating data from 23 bPT interventions that involved a total of 2554 patients with SMI. Our findings offer preliminary evidence supporting the feasibility of bPT treatment for patients with SMI. In examining adherence, our analysis identified it as a crucial factor in exploring and advancing bPT treatment for SMI. However, it is important to note that empirical research in this area is currently limited. The potential positive impact of bPT on adherence in SMI treatment is indicated by the attrition rates reported in the summarized studies. These rates ranged from 0% to 37%, demonstrating favorable outcomes when compared with both guided and unguided technology-based interventions (TBIs). For instance, Musiat et al [64] reported average completion rates of 64% for guided TBIs and 52% for unguided TBIs. When considering attrition rates for mobile health (mHealth) interventions, with a recent meta-analysis reporting an average of 24% [65], the attrition rates found in our studies appear comparable. Nevertheless, the accumulated evidence on the effects of bPT for SMI remains sparse and challenging to extrapolate. Among the 15 studies with a control group design, 4 concluded that bPT interventions were effective compared with control groups. However, 2 of these studies used the same study population, with significantly varied control groups. Additionally, 3 studies investigated patients with affective disorders.

Our feasibility results are in line with earlier systematic evidence on the feasibility of eHealth applications for SMI [66,67], as well as with 2 recent reviews on mHealth for patients with SMI, with a focus on monitoring [18] and disorder management applications [68]. These reviews also established the general acceptance and feasibility of digital interventions in patients with SMI. Our efficacy conclusion is more reflective of the quality of the reviewed research than of the actual subject matter. It primarily focuses on 2 noticeable aspects: First, bPT interventions under scrutiny in the analyzed studies varied greatly in their therapeutic approaches, objectives, digital and face-to-face component features, target groups, and treatment settings, making it challenging to generalize across studies. Second, the studies were heterogeneous regarding research designs, control types, and outcome measures. While these aspects are an outcome of the review’s overall broad scope, they also mirror the current heterogeneity of this field of research.

Despite these conclusions, we remain optimistic about the potential of bPT for the treatment of SMI based on several considerations. On the one side, substantial and increasing evidence supports the effectiveness of face-to-face psychotherapy and psychosocial care for SMI [69-72], although the effects are low to moderate [73,74], and access is often limited due to systemic [75,76] and individual factors [77]. On the other side, the utilization of digital technology and applications is becoming increasingly common among individuals with SMI, and there is ongoing development of digital health interventions [78], including recent advancements in artificial intelligence for SMI treatment [18,79]. However, research also indicates a negative correlation between adherence and the effectiveness of stand-alone TBIs and disorder severity [23,65,80], indicating a fundamental need for human facilitation of digital interventions for SMI [66]. Therefore, bPT, with its concept of enhancing empirically supported psychological treatment with digital content, appears to hold promise for effective, potentially cost-effective, and more accessible treatment for this complex and severely affected patient group. We conclude our review by emphasizing the need for further research in this field.

### A Future Research Agenda on bPT for SMI

We propose several considerations for further research and clinical directions. First, the design of bPT interventions should take into account various contexts of bPT for SMI and consider the advantages and opportunities of different bPT concepts (Multimedia Appendix 4). Using participatory research approaches, as demonstrated by Austin et al [41], can help ensure the creation of appropriate and sustainable treatment solutions based on patients’ needs. Second, it would be advantageous for bPT research to investigate the effects of bPT, giving equal attention to both digital and face-to-face components. This should include the exploration of how these components interact with each other and with additional contexts, such as treatment settings, therapists, and mental health care systems. This would necessitate careful deliberation of suitable control groups. Third, adherence is a critical factor in the effectiveness of TBIs in general [23,65,80], and particularly in the treatment of SMI [12,31,66,81]. Therefore, more research on this topic, including the development of analytical concepts, is required. Fourth, given the multidisciplinary and often long-term nature of SMI treatment, implementation research on bPT for SMI could foster the development of protocols for integrating face-to-face and digital treatment components across varied treatment settings. Lastly, considering the chronic nature of SMI and the increased potential for relapse or decompensation, research on bPT for SMI should encompass the investigation of short- and long-term treatment effects, as well as aspects of treatment safety.

### Limitations

In line with our study design, our literature review was broad in scope, encompassing a wide range of inclusion criteria in terms of study populations, bPT treatment approaches, and study designs. Given the nascent state of the field, this broad approach seemed fitting and allowed for a preliminary overview of the literature. However, the resulting heterogeneity in the findings may reflect this broad approach. Future systematic investigations should consider a more narrow focus to validate our conclusions and build upon the systematic evidence concerning subgroups and categories of bPT treatments for patients with SMI. Additionally, it would be beneficial to include stakeholder and patient perspectives directly in future research endeavors.

Similarly, the inclusion of both controlled and uncontrolled studies may limit the findings of this review. Moreover, due to the absence of quality assessment standards for digital mental health interventions [82], we did not exclude studies based on the potentially inadequate quality of the digital applications utilized. In our review, we only identified 5 studies using mHealth interventions for bPT, which was surprising given the significant advancements and research dynamic in this field. This might suggest that our findings may not generalize to bPT with mHealth.

### Conclusions

The treatment of SMI, which is both prevalent and burdensome for individuals and society, remains challenging [69,75]. In recent decades, psychotherapeutic interventions have emerged as evidence-based first-line treatments, even for patients within this group, yielding mild to moderate effects. Concurrently, the past 25 years have witnessed the development of a broad array of digital mental health interventions. These digital mental health interventions are designed to address treatment gaps, offering affordable, accessible, and scalable alternatives to traditional mental health treatments, and contributing to the overall improvement of mental health care [20,23,80]. In this review, we have summarized the literature on the feasibility, adherence, and effectiveness or efficacy of bPT for patients with SMI. Our findings confirm the feasibility of such treatments for SMI and underline the need for further research to establish the effectiveness of this treatment delivery mode.

Considering that SMI treatment often entails a long history of patient suffering and cost-intensive multidisciplinary care [29,83,84], the further development and implementation of bPT for SMI treatment presents a significant opportunity. This opportunity not only has the potential to further improve the effectiveness and cost-effectiveness of mental health care but is also particularly relevant for this population.

## Appendix

Multimedia Appendix 1 PRISMA-ScR (Preferred Reporting Items for Systematic Reviews and Meta-Analyses Extension for Scoping Reviews) checklist.

Multimedia Appendix 2 Quality assessment.

Multimedia Appendix 3 Data extraction form.

Multimedia Appendix 4 Mapping the field of research on bPT for SMI. bPT: blended psychotherapy; SMI: severe mental illness.

## Abbreviations

APA: American Psychological Association
BDI-II: Beck’s Depression Inventory Second Edition
bPT: blended psychotherapy
CBT: cognitive behavioral therapy
GAD: Generalized Anxiety Disorder (Scale)
mHealth: mobile health
NICE: National Institute for Health and Care Excellence
PHQ-9: 9-item Patient Health Questionnaire
PRISMA-ScR: PRISMA Extension for Scoping Reviews
SMI: severe mental illness
TAU: treatment as usual
TBI: technology-based intervention
