# Supplementary figures and images for: Feasibility, Adherence, and Effectiveness of Blended Psychotherapy for Severe Mental Illnesses: Scoping Review
Source: JMIR Ment Health. 2023 Dec 26;10:e43882. doi: 10.2196/43882 (PMC10777283; doi:10.2196/43882)

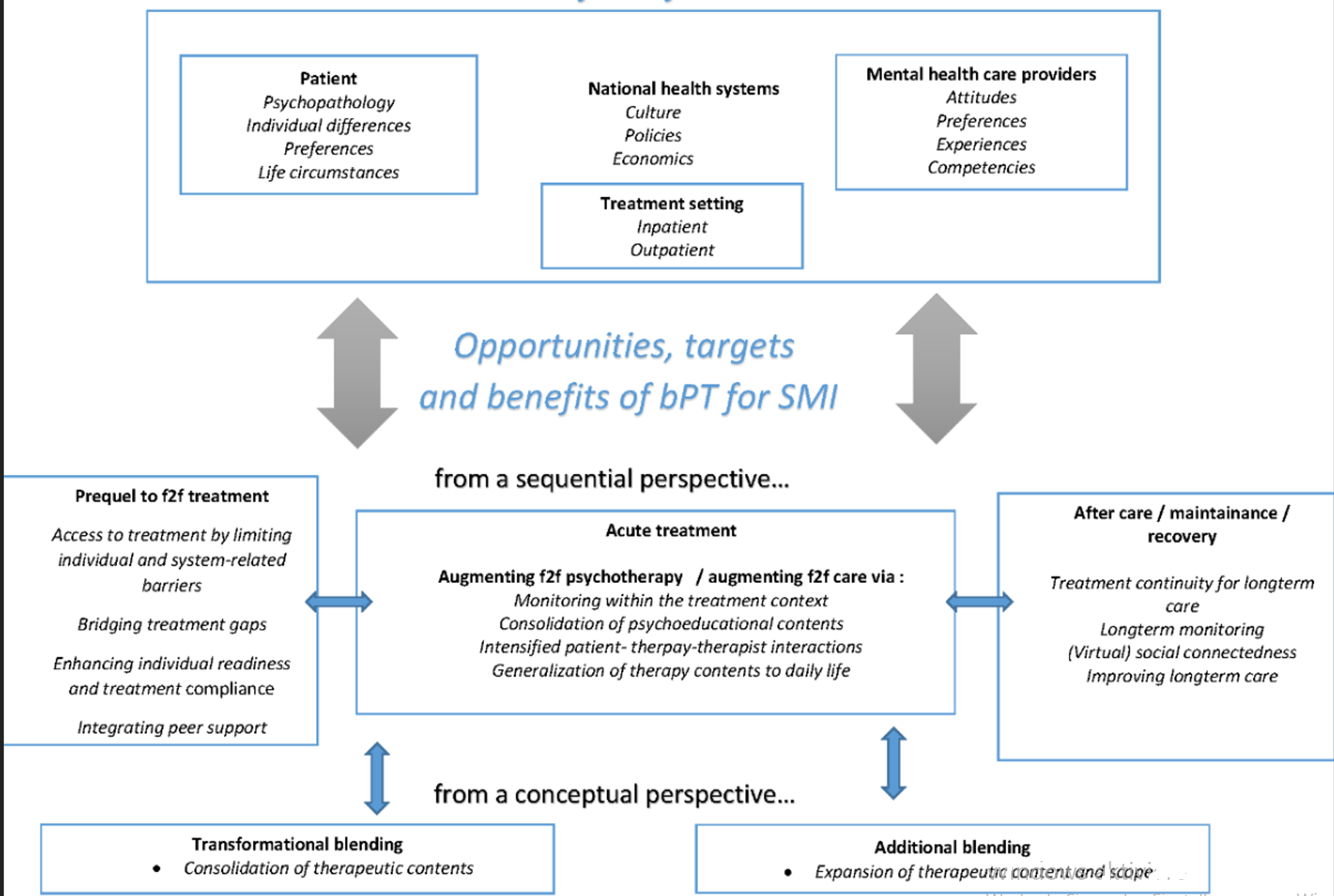

Supplement: Multimedia Appendix 4 [file mental_v10i1e43882_app4.png]
